# Combined Forces Against Bacteria: Phages and Antibiotics

**DOI:** 10.1002/hsr2.70956

**Published:** 2025-07-09

**Authors:** Shima Afrasiabi, Alireza Partoazar, Ramin Goudarzi, Ahmad Reza Dehpour

**Affiliations:** ^1^ Laser Research Center of Dentistry, Dentistry Research Institute Tehran University of Medical Sciences Tehran Iran; ^2^ Experimental Medicine Research Center Tehran University of Medical Sciences Tehran Iran; ^3^ Division of Research and Development Pharmin USA, LLC San Jose California USA; ^4^ Department of Pharmacology, School of Medicine Tehran University of Medical Sciences Tehran Iran

**Keywords:** antibiotic, antimicrobial resistance, bacteriophage, biofilms, MDR, phage–antibiotic synergy

## Abstract

**Background and Aim:**

It is now known that bacteria are highly interactive and exhibit a range of complex cooperative behaviors, including conjugal plasmid transfer, toxins, swarming, drug resistance, toxin production, biofilm development, and other virulence traits. The development of phage–antibiotic synergy (PAS) could be a useful weapon against bacterial infections where antibiotics increase phage replication and antimicrobial activity. This study investigates the therapeutic potential of PAS in combating bacterial infections, focusing on the mechanisms and clinical implications.

**Methods:**

A comprehensive review of recent literature was conducted analyzing studies on PAS, including their effects on biofilm degradation, multidrug‐resistant (MDR) bacteria, and toxin‐producing pathogens. Key factors such as timing, dosing and compatibility of phage‐antibiotic combinations were examined.

**Results:**

PAS showed promising results in various bacterial infections. The combination of phages and antibiotics restored the susceptibility of MDR strains, facilitated the degradation of biofilms and minimized the need for high doses of antibiotics, thereby reducing potential side effects. However, challenges such as the emergence of resistance and antagonistic interactions with certain combinations remain. Key factors influencing the efficacy of PAS include phage and antibiotic dosing, timing of administration, and the physiological state of the bacteria.

**Conclusion:**

PAS has potential applications in the treatment of complex infections. Despite the promising results, further research is essential to standardize protocols, optimize therapeutic combinations and address safety concerns. Clinical trials focusing on phage selection, resistance management and patient‐specific treatments will be crucial for the translation of PAS into clinical practice.

**Abbreviations**
AIs: autoinducers
CA‐MRSA: community–associated methicillin‐resistant *S. aureus*
CR: carbapenem–resistant
ECM: extracellular matrix
LPS: lipopolysaccharides
MDR: multidrug‐resistant
MIC: minimum inhibitory concentration
PAS: phage‐antibiotic synergy
PBPs: penicillin‐binding proteins
PDR: pan‐drug‐resistant
STEC: Shiga toxin‐producing *Escherichia coli*

## 1 Introduction

The increasing emergence of antimicrobial resistance in many regions of the world is a global health and economic threat caused by the excessive and inappropriate use of antimicrobial drugs [1]. Bacteriophages (phages) are viruses that specifically infect bacteria [2]. Phages have two main life cycles. In the “lytic cycle”, viral activity within the bacterial cell leads to cell destruction, while in the “lysogenic cycle”, the phage DNA is incorporated into the bacterial DNA of the host [3]. Phages have unique properties in the elimination of biofilms [4]. Further advantages of phages are their higher specificity than antibiotics, their self‐replication and self‐imitation, their low side effects, and their evolvability [5, 6]. They are effective against multidrug–resistant (MDR) bacteria [6]. Furthermore, they can increase the release of bacterial endotoxins, which can lead to adverse inflammatory responses [7]. In addition, microorganisms that live in a biofilm develop resistance to phages [8]. The ability of bacteria to form biofilms or capsules leads to limited therapeutic efficacy of phages as they have limited access to their target. In addition, phage infection may be limited due to latency and low burst size per infected cell, low metabolic activity, or dormancy of bacteria in biofilm mode [9].

**Figure 1:**
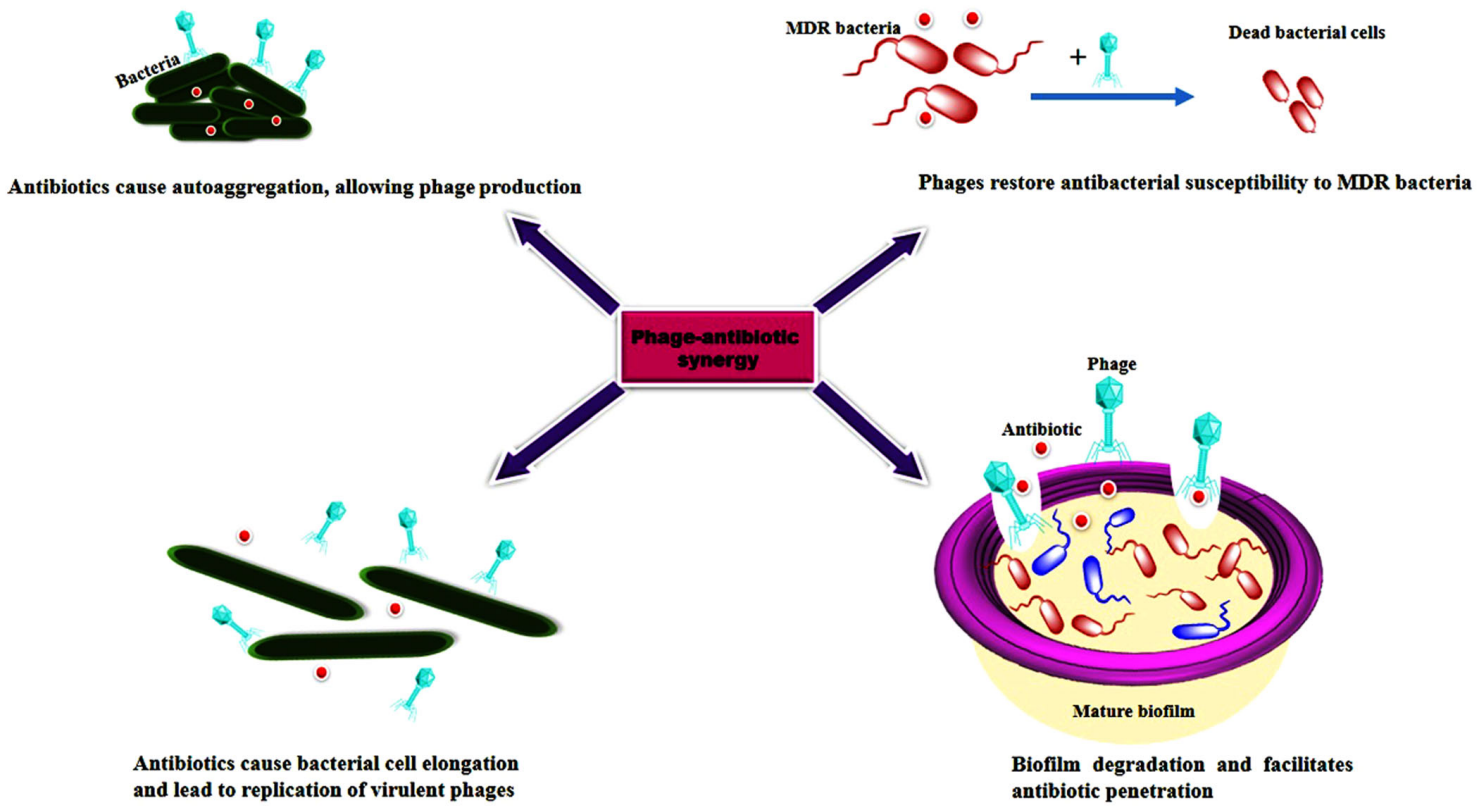
The synergy between phages and antibiotics.

The phenomenon of phage–antibiotic synergy (PAS) is described by the fact that administration of sublethal concentrations of antibiotics in phage‐infected bacteria significantly compromises the integrity of the cell wall, leading to an increase in phage plaque activity, size, and cell lysis [10, 11]. Phages as adjuvants retain their potential antibacterial activity when combined with antibiotics [10]. The synergy between phages and antibiotics offers several advantages, as shown in Figure 1 [12].

However, this combination can lead to the emergence of drug‐resistant phenotypes due to competitive release [13]. In addition, antibiotics could potentially interfere with aspects of bacterial metabolism required for phage infection. For these reasons, the effects of PAS should be investigated to avoid antagonistic effects [6]. Therefore, in this review, the most important studies on the PAS against bacterial infections are investigated. This would help to optimize antibacterial strategies.

## 2 PAS and Biofilm Removal

Phages and antibiotics often have different effects on the same species, depending on the lifestyle of the bacterial cells [14]. Biofilms are structured bacterial communities embedded in extracellular matrix (ECM), inherently resistant to external stress, antibiotic therapies, and host defenses. Therefore, biofilms represent a significant clinical obstacle to the successful treatment of infectious diseases [15]. Biofilm formation can be explained in a sequential manner that includes initial attachment, maturation, and dispersal. Quorum sensing regulates gene expression based on cell density, influencing biofilm formation and virulence [16].

Biofilms differ significantly from planktonic bacteria in structure and behavior, particularly in their heightened resistance to antibiotics [17, 18, 19, 20]. Several factors contribute to the high tolerance of biofilms to antimicrobial agents, for example, physiological changes, a slow growth rate, difficult penetration of antibiotics into biofilm structures, a large genetic diversity of bacterial population, the existence of “persister” cells and the multi‐species biofilms found in this context [21, 22].

*Pseudomonas aeruginosa* is considered a clinically important bacterium in PAS. This opportunistic pathogen is frequently found in cystic fibrosis, respiratory, urinary tract, and hospital–acquired infections. *P. aeruginosa* colonizes heavily in acute and chronic infections and is resistant to conventional antibiotic therapies [23]. Chaudhry et al. showed that treatment of *P*. *aeruginosa* PA14 biofilms with ciprofloxacin, ceftazidime, colistin, gentamicin, and tobramycin alone had only moderate anti‐biofilm efficacy. However, when co‐administered with the two phages NP1 and NP3, synergism was observed at onefold and eightfold minimum inhibitory concentration (MIC) for ceftazidime and only at MIC for ciprofloxacin. No synergy between phage and colistin or gentamicin was observed. Timing and dosage significantly affect PAS efficacy. A remarkable synergistic effect was obtained with the addition of tobramycin or gentamicin 24 h after phage treatment. The tobramycin results may seem counterintuitive. However, the combination of tobramycin (1 MIC) and phage resulted in the strongest overall kill; the addition of phage to 8 MIC of this antibiotic did not increase the kill beyond that of the drug alone. Conversely, the successive addition of ceftazidime or ciprofloxacin did not produce better results than the simultaneous use of these agents. Therefore, the dose and timing are crucial for a successful combination use [24].

While phages can act synergistically with antibiotics, the combination of phage and antibiotic may not be effective. Danis‐Wlodarczyk et al. reported that the giant phage KTN4 in combination with colistin showed no synergistic effects against *P. aeruginosa* PAO1, while both agents alone were effective in reducing biofilm. The authors postulated that colistin could limit the spread of phages because it destabilizes the cell membrane [25]. The combination of tobramycin and the phage PB‐1 also does not lead to a further reduction of the *P. aeruginosa* PAO1 biofilm. However, the combination reduces the occurrence of phages and antibiotic‐resistant bacteria [26]. Many phages encode enzymes such as depolymerases that degrade ECM, which facilitates phage penetration and promotes biofilm destruction. In *P. aeruginosa*, for example, phages such as phiKZ and EL produce alginate lyases that degrade the alginate‐rich matrix characteristic of biofilms, promote the spread of antibiotics and enhance synergistic effects [27, 28].

Biofilm formation is an important cause of infections associated with *Escherichia coli*. Bacterial biofilms are now more difficult to treat due to the increased occurrence of antibiotic resistance markers in the biofilm [29]. Ryan et al. found that the use of PAS for the phage T4 (Myoviridae, T4virus) increased the size of the T4 burst by shortening the latency of T4. This phage plus cefotaxime achieved remarkably stronger biofilm inhibition of *Escherichia coli* compared to biofilm treatment with cefotaxime alone. Lower antibiotic doses were effective when combined with phages to eradicate the *E. coli* biofilm. Therefore, reducing the therapeutically effective amount of antibiotics with phages could be a useful strategy to minimize the side effects of antibiotics in vivo [30]. Coulter et al. have shown that with the same strain and phage and using tobramycin, the survival rate of *E. coli* cells within biofilm decreased to almost zero [26]. The T4 phage has been shown to express tail‐associated depolymerases that degrade capsular polysaccharides and biofilm matrix components, resulting in improved phage access and increased efficacy in combination with antibiotics [31].

*Staphylococcus aureus* remains an important cause of both community and hospital‐acquired infections [32, 33]. Increasing penicillin resistance, followed by the spread of resistance to oxacillin, methicillin, nafcillin, and now even vancomycin, has made the treatment of staphylococcal disease a global challenge [32]. Kumaran et al. investigated the potential of phages to enhance antibiotic activity at lower antibiotic concentrations for the inhibition of *S. aureus* biofilms. The results showed that each antimicrobial alone caused a minimal reduction in biofilm, but a 3 log_10_ reduction was achieved when phage treatment preceded the antibiotics. Furthermore, when *S. aureus* biofilms were treated simultaneously with cefazolin, vancomycin, linezolid, or tetracycline, antagonism between the two agents was seen for all antibiotics used [34]. In addition, Chaudhry et al. showed that phages can rapidly multiply in the dense bacterial environmental of the biofilm when biofilms are pretreated with phages before antibiotic treatment, resulting in high phage titer and destruction of the biofilm matrix [25]. Therefore, the order of treatment is important and, if reversed, can lead to antagonistic effects on the outcome of biofilm control. The reduction in anti–biofilm activity observed at higher concentrations may be due to the lower bacterial density limiting new phage infections and thus reducing the overall anti‐biofilm effect [34]. Akturk et al. stated that PAS was only observed with simultaneous combinations when antibiotics were used at the MIC level. Surprisingly, high antibiotic concentrations may reduce PAS efficacy due to phage inhibition. This process could be related to the phenomenon of inhibition of phage replication by DNA and protein synthesis inhibitors. Their work involved both biofilm formation inhibition and mature biofilm treatment [35]. On the other hand, phage dose is also an important factor in PAS. Both low–dose and medium–dose phages can produce significant additive antibacterial effects. The synergy of high–dose phages decreases over time. In fact, high–dose phages have induced stress on bacteria, leading to increasing bacterial resistance [36].

## 3 PAS and Toxin‐Producing Bacteria

Shiga toxin‐producing *E. coli* (STEC) are capable of producing at least one member of a class of potent cytotoxins known as Shiga toxin [37]. STEC strains have been associated with life‐threatening infections and the ability to form biofilms [38]. Zhang et al. reported that the risk of using phage‐inducing antibiotics, such as fluoroquinolones to treat STEC should be considered, probably due to the induction of ST‐converting prophages from one *E. coli* to another and the enhancement of the movement of virulence factors caused by antibacterial agents [39]. Necel et al. showed that the phage vB_Eco4‐M7 effectively prevents biofilm formation in STEC. However, the greatest reduction was observed when phage infection occurred before treatment with ciprofloxacin or rifampicin (sixfold vs. 2–3‐fold biofilm reduction). This study showed that ciprofloxacin acted as an efficient classical inducer, whereas prophage induction could not be detected in rifampicin‐treated STEC cells [40]. Easwaran et al. investigated the ability of the phage ΦEcSw in combination with chloramphenicol or kanamycin on *E. coli* O157:H7, which causes urinary tract infections and bloody diarrhea in humans [41, 42]. The results of this study were promising and ΦEcSw has the potential to cure infected animals. However, the effect of this combination on the biofilm and the effect of the antibiotics on the induction of ST‐converting prophages have not been investigated [42].

## 4 PAS and MDR Bacterial Infections

**Figure 2:**
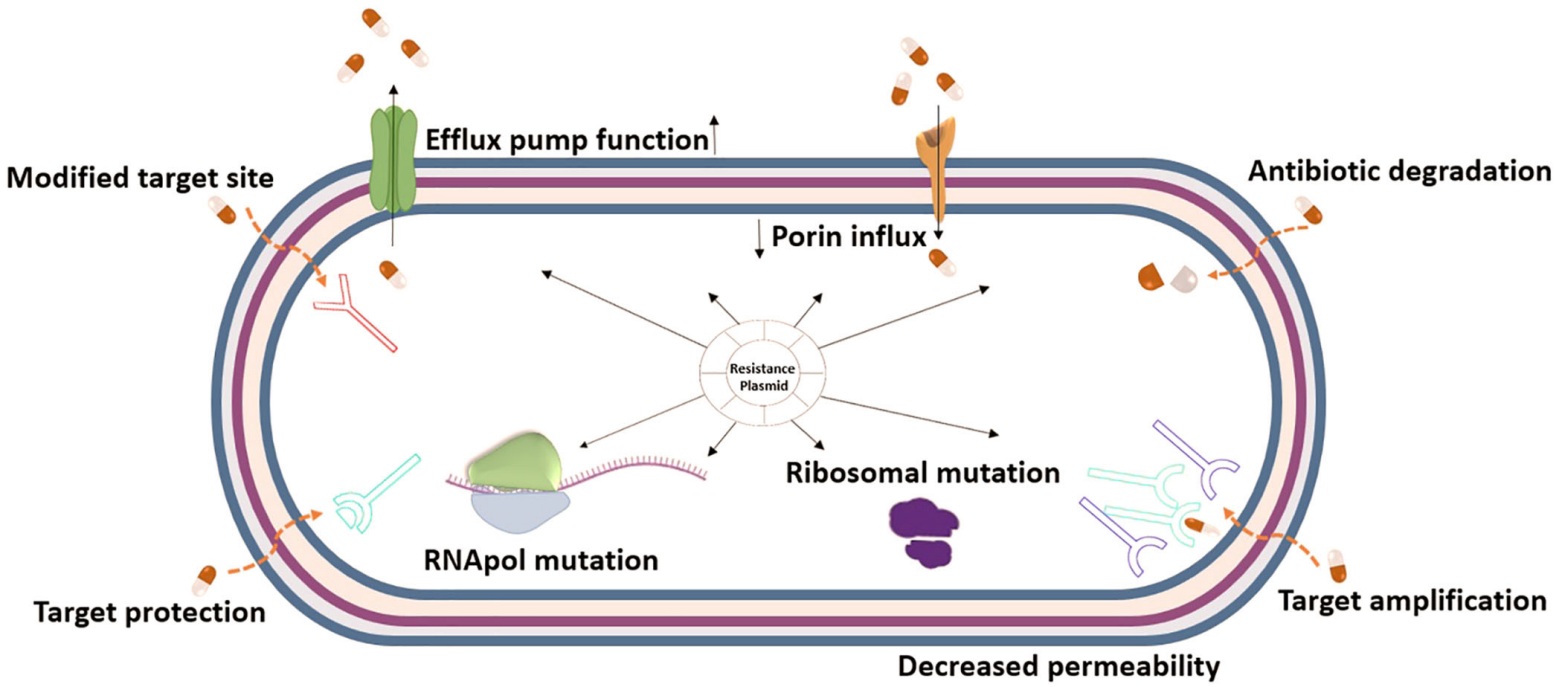
Mechanism of antimicrobial resistance.

ESKAPE pathogens, which include *Enterococcus faecium*, *S. aureus*, *Klebsiella pneumoniae*, *A. baumannii*, *P. aeruginosa*, and *Enterobacter* spp., pose a significant global threat to human health due to the acquisition of antibiotic resistance genes. MDR strains are described as resistant at least one agent in three or more antimicrobial classes [43, 44]. Infections with MDR are associated with inadequate or delayed antimicrobial therapy and may result in poorer treatment outcomes [45]. The most important mechanisms of resistance are shown in Figure 2 [46].

Kamal and Dennis found that the PAS is not affected by the antibiotic resistance status of the target cell and that this combination can still be used to treat cells that are known to be resistant to a variety of antibiotic treatments [11]. Even for MDR bacteria such as *Burkholderia cenocepacia*, *S. aureus*, or *P. aeruginosa*, several synergies have been reported as successful [11, 32, 47]. Engeman et al. found that the use of the PAM2H phage cocktail instead of monotherapy increased the susceptibility of MDR *P. aeruginosa* to ceftazidime, meropenem, gentamicin, and ciprofloxacin [48]. PAS with different mechanisms leads to simultaneous eradication of both antibiotic–susceptible and phage‐susceptible populations, resulting in a very small population of bacteria that is both antibiotic‐ and phage‐insensitive [49].

A study by Lin et al. found that the PAS in *P. aeruginosa* is antibiotic and/or strain‐dependent. In the clinical strain *P. aeruginosa* FADD1‐PA001, the synergistic effect of podophage PEV20 in combination with ciprofloxacin, amikacin, or colistin was demonstrated. In the case of *P. aeruginosa* strain JIP865, synergies also occurred between the phage PEV20 and amikacin, ciprofloxacin, or tobramycin. However, complete inhibition of bacterial growth is only achieved with the phage PEV20 plus ciprofloxacin. In contrast, no synergistic effect was observed when the phage PEV20 was combined with the above‐mentioned antibiotics in strain 20844n [50].

*Acinetobacter baumannii* is known to be one of the most common infections in humans due to its intrinsic or acquired resistance and its potential for long‐lasting persistence in the environment [51]. Approximately 50% of clinical infections with *A. baumannii* are caused by MDR strains [52]. In the study by Jansen et al., the T4‐like phage KARL‐1, which infects eight MDR strains of *A. baumannii*, showed marked synergy with meropenem or colistin, while ciprofloxacin did not support the activity of the phage [53]. In contrast, treatment with antibiotics alone had little effect against MDR *A*. *baumannii* strains [51]. *A*. *baumannii* may exhibit a variety of resistance mechanisms, including alterations in penicillin‐binding proteins (PBPs), beta‐lactamase‐induced hydrolysis, changes in porin proteins leading to decreased outer membrane permeability, and efflux pump activity that further decreases antibiotic concentration in the bacterial cell [54]. Wintachai et al. found that the combination of the phage vWU2001 with colistin was a much more effective agent against the multiplication of carbapenem–resistant (CR) *A. baumannii* and prevented the emergence of bacterial resistance. PAS led to rapid phage maturation, which enabled forced cell lysis. The cluster structure of the bacterial cells in the presence of colistin allowed the phages to move on the surface of neighboring cells, which increased the efficiency of phage infection [55].

*Klebsiella pneumoniae* has been identified as one of the potential opportunistic pathogens that can cause nosocomial and community‐acquired infections [56]. *K. pneumoniae* can behave as a pan–drug–resistant (PDR) bacterium. The spread of PDR *K. pneumoniae* species is a serious challenge worldwide [57]. The emergence of CR *K. pneumoniae* has exacerbated the dilemma of antimicrobial use and triggered the need for alternative therapies [58]. Wang et al. found that phage vB_KpnM_P‐KP_2_ eliminated the bacteria in the lungs of mice and enhanced the therapeutic effect of gentamicin, resulting in inhibition of inflammation development and preventing the occurrence of further CRK [59]. Pacios et al. found that host cell lysis by phage vB_KpnM‐VAC13 is regulated by recognition of its receptor, while DNA strand cross‐linking is induced by mitomycin C. Thus, a multitarget effect is the main cause of this synergism [60].

*Enterococcus faecalis* has become the third most common nosocomial pathogen worldwide [61]. The problem with this infection is the fact that enterococci are intrinsically resistant to many antimicrobial agents and only a few antibiotics are effective against them [62]. Shlezinger et al. demonstrated that the viability and biomass of vancomycin‐resistant *E. faecalis* were reduced after exposure to the combination of the phage EFLK1 and vancomycin. Furthermore, in the presence of the phage, a lower amount of vancomycin is required to overcome *E. faecalis* [63].

## 5 Phage Derived Proteins and Enzymes

In addition to intact phages, phage‐derived proteins and enzymes—such as endolysins, depolymerases, and holins—have emerged as promising therapeutic tools, either alone or in combination with antibiotics. Endolysins have attracted considerable attention as potent antibacterial agents, particularly due to their ability to lyse bacterial cell walls in a highly specific and efficient manner [64]. Their use has been extended by combination strategies with conventional antibiotics to enhance antibacterial efficacy and combat MDR pathogens. Thummeepak et al. demonstrated the synergistic effect of endolysin LysABP‐01 with colistin against *A. baumannii*, reducing the MIC of LysABP‐01 by 32‐fold and that of colistin by eightfold [65]. A similar synergy was observed against vancomycin‐intermediate *S. aureus* (VISA) with MV‐L, an anti‐staphylococcal endolysin, in combination with glycopeptides such as vancomycin and teicoplanin. The enhanced lytic activity was attributed to improved antibiotic penetration facilitated by partial degradation of the thickened cell wall [66]. In addition, SAL200, a recombinant phage endolysin, restored the efficacy of nafcillin and vancomycin both in vitro and in mice and Galleria mellonella infection models [67]. In another study, Letrado et al. reported a strong synergy between endolysin Cpl‐711 and β‐lactam antibiotics against *Streptococcus pneumoniae* [68], while Becker et al. showed that the combination of endolysin Lys K with lysostaphin significantly improved bactericidal outcomes against *S. aureus* [69]. Moreover, a previous study found that coadministration of cysteine/histidine‐dependent amidohydrolase/peptidase (CHAP) and amidase catalytic domains of endolysin Lys K with vancomycin resulted in an eightfold reduction in the MIC of vancomycin [70]. Taken together, these results emphasize the therapeutic potential of endolysins, not only as stand‐alone agents, but also as enhancers of existing antibiotic regimens.

Yang et al. showed that the combination of Dep37, a phage‐derived depolymerase, and kanamycin was significantly more effective than Dep37 or kanamycin alone in the treatment of carbapenem‐resistant *K. pneumoniae* biofilms. Dep37 specifically targets and cleaves the glycosidic bonds within the capsule structure, leading to a reduction in capsule thickness and making the bacteria more susceptible to recognition and elimination by the host immune system. This process increases the susceptibility of the bacterium to antibiotic therapy as the capsule, which normally provides a barrier to antibiotic penetration, is compromised [71]. Latka and Drulis‐Kawa reported that the combined use of depolymerase with ciprofloxacin and phage was effective against biofilms of MDR *K. pneumoniae* [72]. Wu et al. showed that the phage depolymerase Dep42 was able to enhance the activity of polymyxin against *K. pneumoniae* biofilms [73]. Bansal et al. evaluated the synergistic effect of the phage KPO1K2 or bacterial depolymerase in combination with gentamicin against biofilms formed by *K. pneumoniae* strain B5055. The combination of phage‐derived depolymerase and gentamicin led to a significant reduction in bacterial cell counts compared to the control group. In contrast, gentamicin alone showed a less pronounced effect [74].

Holins—membrane‐penetrating proteins encoded by phages—are responsible for hole‐forming proteins in the bacterial cytoplasmic membrane that allow endolysins to access their peptidoglycan substrate [75]. Notably, holins have also shown direct antibacterial potential when combined with antibiotics. Zhang et al. showed significant synergistic effects of the holin HolSSE1 with various antibiotics against *Shigella* spp. and *S. aureus* biofilms. HolSSE1 in combination with erythromycin resulted in the highest biofilm removal rate from *S. dysenteriae* 1.1869, while the combination with polymyxin B sulfate was effective against *S. flexneri* 1.1868. Similarly, HolSSE1 in combination with cephalothin improved the removal of *S. flexneri* 1.10599 biofilms. These combinations outperformed antibiotics alone at ½ MIC, highlighting the importance of holins in increasing membrane permeability, thereby enhancing the efficacy of conventional antimicrobials [76].

## 6 Recent Advancements and Barriers in Clinical Trials

Recent clinical research provides promising insights into the application of PAS in the treatment of MDR infections. Bao et al. conducted a study demonstrating the synergistic effect of a *K. pneumoniae* phage cocktail in combination with a non‐active antibiotic in a clinical setting. The study describes the successful recovery of a patient suffering from recurrent urinary tract infections (UTIs) after treatment with five lytic phages and the antibiotic trimethoprim‐sulfamethoxazole (TMP/SMX). Notably, all *K. pneumoniae* strains isolated from the patient UTI showed complete resistance to TMP/SMX [77]. In the study by Law et al. phage therapy was used in combination with antibiotics to treat an MDR *P. aeruginosa* infection in a patient with cystic fibrosis. Although the patient's condition did not respond to conventional antibiotic treatments, PAS resulted in significant clinical improvement, including reduced bacterial load and resolution of the infection [78]. Pirnay et al. investigated the results of a retrospective observational study of 100 consecutive cases of personalized phage for the treatment of difficult‐to‐treat infections. The study showed that PAS can lead to significant clinical improvement, with clinical improvement achieved in 77.2% of infections and eradication of bacteria in 61.3%. The results also suggest that the simultaneous use of antibiotics significantly increases the likelihood of bacterial eradication [79]. These studies underscore the emerging potential of PAS in clinical applications, while also highlighting the necessity for further large‐scale trials to confirm these findings.

## 7 Disadvantages, Limitations, and Future Directions

While PAS is very promising for the treatment of infections, there are also some potential drawbacks and challenges that need to be considered. Similar to antibiotics, bacteria may develop resistance to phages via mutation of the receptor sites targeted by the phage, restriction‐modification systems, or the production of extracellular substances that prevent the phage from binding. Over time, the emergence of phage‐resistant bacteria could limit the long‐term efficacy of PAS [80]. Phages usually have narrow host ranges, limiting their use as broad‐spectrum agents [81]. Certain antibiotics can impair or even inactivate phages, especially if they target bacterial processes that are also important for phage replication, such as cell wall synthesis [82]. Regulatory, safety, and microbiome impact concerns complicate clinical phage use [83, 84]. Phages are less stable than antibiotics, facing challenges in storage and environmental sensitivity [85]. Biofilm‐associated bacteria act as a physical barrier and impede phage movement and penetration [86]. Phage‐induced lysis of bacteria could lead to the release of endotoxins, which can trigger an inflammatory response in the host [87].

There are a number of limitations to the studies conducted to date. Many PAS studies lack standardization, overlook host genetics or prophages, and yield inconsistent results due to differing methodologies [88]. There are not many studies that elucidate the mechanism of PAS, and clinical studies in this area should be continued.

This study suggests the following key elements for future standardized protocols in PAS research: Development of a repository of phages tested against clinically relevant bacterial strains. Standardized methods for evaluating the efficacy of PAS, such as phage‐antibiotic checkerboard assays or time‐kill curve analyzes. Guidelines for patient‐specific therapy, including dosing optimization and timing of phage and antibiotic administration. Implementation of routine genomic screening of phages to ensure that no lysogenic or toxin‐related genes are present. Standardize protocols for testing the efficacy of phages against specific bacterial strains using quantitative methods such as plaque assays and time‐kill curves. Adopt uniform protocols for checkerboard assays to quantify phage‐antibiotic interactions and determine the Fractional Inhibitory Concentration Index. Include biofilm degradation assays using consistent parameters such as incubation time, bacterial density, and matrix composition to evaluate PAS in biofilm‐associated infections. Establish guidelines for animal models that accurately mimic human infections, including standardized routes of infection, phage dosing regimens, and endpoints for evaluating efficacy. Propose harmonized frameworks for selecting PAS based on patient‐specific data, including bacterial susceptibility profiles and immune system interactions. Develop universal reporting criteria for clinical trials, including phage pharmacokinetics, resistance emergence, and patient outcomes.

## 8 Managing Risks (Emergence of Phage or Antibiotic Resistance and Potential Antagonistic Interactions)

The use of phage mixtures that target multiple bacterial receptors reduces the likelihood of developing resistance to all phages in the cocktail [48, 89, 90]. Switching between different phages or between phages and antibiotics minimizes the selection pressure on the bacteria to develop resistance [49]. PAS often allows for a lower dose of antibiotics while maintaining efficacy, reducing the risk of resistance [30]. Phage therapy can restore bacterial sensitivity to previously ineffective antibiotics, providing more therapeutic options [11, 26]. Avoiding antibiotics that interfere with phage activity, such as those that inhibit bacterial replication required for phage production (e.g., protein synthesis inhibitors), may attenuate antagonism [25]. The administration of phages before or after the administration of antibiotics, depending on the reaction of the bacteria, can help to avoid negative interactions [24, 34]. Screening phages for lysogenic and toxin‐related genes by whole genome sequencing ensures that the selected phages are strictly lytic and do not enhance toxin production [91]. Encapsulation or modification of phages can minimize immune response and increase efficacy [92]. Table 1 also provides further examples of phages, antibiotics and potential synergistic combinations against bacterial infections. PAS and other virulence traits, along with supporting references, are provided in the Supporting Information [117, 118, 119, 120, 121, 122, 123, 124, 125, 126, 127, 128, 129, 130, 131, 132, 133, 134, 135, 136, 137].

**Table 1:** Additional examples of phage‐antibiotic synergy (PAS) against bacterial infections.

Phage (s)	Antibiotic (s)	Bacteria	Synergistic effects	Ref.
PEV20	Ciprofloxacin	*Pseudomonas aeruginosa*	Biofilm reduction	[93]
Sb‐1	Teicoplanin	MRSA	Biofilm reduction	[94]
Cocktail of six phages	Amikacin/Meropenem	*Pseudomonas aeruginosa*	Biofilm reduction	[95]
Φ4_ZP1	Meropenem	*Pseudomonas aeruginosa*	Biofilm reduction	[96]
Φ14_OBG	Tobramycin	*Pseudomonas aeruginosa*	Biofilm reduction	[96]
KPP22	Piperacillin/Ceftazidime	*Pseudomonas aeruginosa*	Biofilm reduction	[97]
SAP‐26	Rifampicin	*Staphylococcus aureus*	Biofilm reduction	[98]
SATA‐8505	Cefazolin/Vancomycin	*Staphylococcus aureus*	Biofilm reduction	[34]
EPA1	Gentamicin/Ciprofloxacin/Meropenem	*Pseudomonas aeruginosa*–*Staphylococcus aureus*	Biofilm reduction	[35]
vB_1086	Meropenem	*Klebsiella pneumoniae*	Biofilm reduction	[99]
KPO1K2	Ciprofloxacin	*Klebsiella pneumoniae*	Biofilm reduction	[100]
vB8388	Gentamicin	*Klebsiella oxytoca*	Biofilm reduction	[101]
vB_PmiS‐TH	Ampicillin	*Proteus mirabilis*	Biofilm reduction	[102]
YC#06	Chloramphenicol/Minocycline/Imipenem/Cefotaxime	MDR *Acinetobacter baumannii*	Biofilm reduction	[36]
Cocktail of 3‐ or 4‐phage	Ampicillin/Daptomycin	MDR *Enterococcus faecium*	Biofilm reduction	[89]
φAB182	Colistin/Polymixin B/Ceftazidime/Cefotaxime	MDR *Acinetobacter baumannii*	Biofilm reduction	[103]
LKD16	Carbenicillin/Gentamicin/Trimethoprim	*Pseudomonas aeruginosa*	Bacterial reduction	[104]
KS12/KS14	Meropenem/Ciprofloxacin/Tetracycline	*Burkholderia cepacia*	Bacterial reduction	[11]
BFC 1.10.	Ceftazidime/Avibactam	MDR *Pseudomonas aeruginosa*	Bacterial reduction	[105]
SA5	Gentamicin	*Staphylococcus* aureus	Bacterial reduction	[106]
LUZ7	Streptomycin	*Pseudomonas aeruginosa*	Bacterial reduction	[107]
Pf3	Tetracycline/Gentamicin/Carbenicillin/Chloramphenicol	*Pseudomonas aeruginosa*	Bacterial reduction	[108]
Sb‐1	Oxacillin	MRSA	Bacterial reduction	[109]
SALSA	Ampicillin/Sulbactam	*Serratia marcescens*	Bacterial reduction	[110]
ØABP‐01	Colistin	*Acinetobacter baumannii*	Bacterial reduction	[111]
P22	Ceftriaxone/Ciprofloxacin	MDR *Salmonella typhimurium*	Bacterial reduction	[112]
vABWU2101	Tigecycline	MDR *Acinetobacter baumannii*	Bacterial reduction	[113]
MR‑5	Linezolid	MRSA	Bacterial reduction	[114]
Ab105‐2phiDCI	Meropenem	MDR *Acinetobacter baumannii*	Bacterial reduction	[115]
vB_KpnM‐VAC13	Mitomycin C/Imipenem	MDR *Klebsiella pneumoniae*	Bacterial reduction	[60]
Cpl‐711	Amoxicillin/Cefotaxime	MDR *Streptococcus pneumoniae*	Bacterial reduction	[116]

Abbreviations: MDR, multi‐drug resistant; MRSA, methicillin‐resistant *Staphylococcus* aureus.

## 9 Conclusion

Recent studies indicate that the timing and concentration of phage and antibiotic administration significantly influence therapeutic outcomes. Recent studies have shown that combining multiple phages (phage cocktails) and alternating phage‐antibiotic combinations can help delay the development of resistance. Toxin production, as observed in STEC, can be reduced by selecting purely lytic phages that do not carry prophage‐related genes. Screening phages for virulence and toxin‐associated genes using whole genome sequencing is an essential step to ensure safety. Under the combined pressure of phages and antibiotics, some important virulence factors of the host bacteria are lost or reduced, which are related to bacterial toxicity, biofilm formation, and drug resistance. Currently, we still need a deeper understanding of the molecular basis and the exact mechanisms that determine phage‐antibiotic interactions. Therefore, it is currently a challenge to predict the ideal combination for a bacterial pathogen. Nevertheless, the promising results obtained so far indicate that further testing of PAS is an endeavor that is likely to be an effective treatment against bacterial virulence factors in the future.

## Author Contributions

**Shima Afrasiabi:** conceptualization, writing – original draft, writing – review and editing, supervision, project administration. **Alireza Partoazar:** investigation, validation, visualization. **Ramin Goudarzi:** investigation, validation, visualization. **Ahmad Reza Dehpour:** investigation, validation, visualization.

## Ethics Statement

The authors have nothing to report.

## Conflicts of Interest

The authors declare no conflicts of interest.

## Transparency Statement

The lead author Shima Afrasiabi affirms that this manuscript is an honest, accurate, and transparent account of the study being reported; that no important aspects of the study have been omitted; and that any discrepancies from the study as planned (and, if relevant, registered) have been explained.

## Supporting information

Supporting material 2.

## Data Availability

All data are available in the main text. All authors have read and approved the final version of the manuscript. Shima Afrasiabi had full access to all of the data in this study and takes complete responsibility for the integrity of the data and the accuracy of the data analysis. No data set available as no new data were generated.
