# Supplementary material for: Combined Forces Against Bacteria: Phages and Antibiotics
Source: Health Sci Rep. 2025 Jul 9;8(7):e70956. doi: 10.1002/hsr2.70956 (PMC12239521; doi:10.1002/hsr2.70956)
Supplement: Supplementary file 1 — Supporting material 2. [file HSR2-8-e70956-s001.docx]

**Supplementary material**

**Combined Forces Against Bacteria: Phages and Antibiotics**

**Shima Afrasiabi^1, *^, Alireza Partoazar^2^, Ramin Goudarzi^3^, Ahmad Reza Dehpour^2,4^**

^1^Laser Research Center of Dentistry, Dentistry Research Institute, Tehran University of Medical Sciences, Tehran, Iran

^2^Experimental Medicine Research Center, Tehran University of Medical Sciences, Tehran, Iran

^3^Division of Research and Development, Pharmin USA, LLC, San Jose, CA, USA

^4^Department of Pharmacology, School of Medicine, Tehran University of Medical Sciences, Tehran, Iran

^*^Correspondence:

^Shima Afrasiabi,^

^Ph.D., Assistant Professor of Medical Bacteriology, Laser Research Center of Dentistry, Dentistry Research Institute, Tehran University of Medical Sciences, Tehran, Iran.^

^Tel: +98-21-8838-4331^

E-mail: shafrasiabi**@sina.tums.ac.ir**

**PAS and other virulence traits**

Phage–resistant phenotypes that exhibited mutations in genes leading to alteration of bacterial surface components such as lipopolysaccharides (LPS), outer membrane, capsule, teichoic acid, capsules, type IV pili, efflux pumps, and siderophore receptors as known virulence factors [117,118]. Phage resistance could lead to reduced virulence or re-sensitization to antibiotics [117]. Capparelli et al. showed that phage-resistant *S. aureus* A172 exhibited reduced growth rate, under expression of several important genes, capsular polysaccharide production, loss of terminal GlcNA, and altered teichoic acid structure compared to the parental strain [119].

LPS is a unique glycolipid found on the outer surface of most Gram-negative bacteria that acts as a primary docking site for phages, allowing them to target phages [120]. LPS consists of three parts: lipid A, core, and O-antigen [121]. Lipid A serves as the hydrophobic part of the outer membrane [122]. The core oligosaccharide and the O-antigen help the bacteria to defend themselves against antibiotics, the complement cascade and other external influences [121]. The O antigen or core is recognized by phages. In general, O antigens differ greatly between Gram-negative bacteria, while the core is highly conserved [123]. LPS forms a tight permeability barrier for hydrophobic antibiotics, and strains expressing full-length LPS exhibit intrinsic resistance [124]. Nesper et al. found that the full-length O1 antigen of *Vibrio cholerae* acts as a specific receptor for phage K139. *V. cholerae* O1 El Tor mutants that exhibit spontaneous resistance to phage K139 synthesize LPS-deficient (O antigen-negative) [125]. The presence of LPS–binding phage can cause cells to develop phage resistance by losing or modifying LPS, leading to antibiotic sensitivity. Tamaki et al. found that T4-resistant strains lacking both the phosphate groups and the outer core exhibited increased sensitivity to hydrophobic novobiocin. Therefore, the phosphate groups play an important role in the stabilization of LPS for resistance to novobiocin [126]. Liu et al. showed that phage-resistant mutations that occurred in *rfa* gene clusters resulted in an altered LPS structure that made *E. coli* more susceptible to colistin [127]. In another study, Davis et al. found that the phage E79 and the antibiotic aztreonam showed PAS on *P. aeruginosa* PA01. LPS is a receptor for the lytic phage E79, which attaches to bacterial host cells for infection. A maximal effect is observed on the diameter of phage E79 plaques, which form large plaques in the presence of aztreonam lysine [128].

The bacterial capsule and the outer membrane serve as primary and secondary phage receptors for phab24, respectively. In particular, knock-out of the *gtr9* gene that mediates phage resistance, a gene for capsule biosynthesis, increased sensitivity to colistin. Indeed, the absence of a barrier facilitates the diffusion of colistin across the membrane [117]. Similarly, Altamirano et al. illustrated the emergence of resistance of *A. baumannii* AB900 to phage ΦFG02 by loss of the capsule, resulting in reduced fitness *in vivo* and re-sensitization to ceftazidime [129]. In addition, type IV pili and twitching motility are an important component of *P. aeruginosa* biofilm formation [130]. Chibeu et al. showed that the loss of type IV pili led to resistance to the phage ФKMV and reduced twitching motility [131]. Capparelli et al. found a similar relationship between the acquisition of phage resistance and loss of virulence in *Salmonella enterica* [132].

Some bacteria have developed resistance to antibiotics by expressing multidrug efflux pumps that reduce drug concentrations to subtoxic levels, thus affecting both intrinsic and acquired resistance [133]. On the other hand, there are bacteria that are resistant to phages that utilize efflux pumps as receptors, resulting in reduced pump activity and increased susceptibility to antibiotics [134]. Therefore, reduced protein expression of bacterial drug efflux pumps occurs after combined treatment [49]. Chan et al. found that the phage OMKO1 of *P. aeruginosa* utilizes the multidrug efflux pump mexA-mexB- outer membrane porin M (OprM). The deactivated OprM increases phage resistance and alters the proteins of the efflux pump. This leads to a sensitivity to antibiotics such as tetracycline, erythromycin, ceftazidime, and ciprofloxacin [135]. In this context, Petsong et al. found that combined treatment with ciprofloxacin and phage P22 can decrease the expression of genes related to the efflux pump (*acrA*, *acrB*, and *tolC*) and genes related to the outer membrane (*ompC*, *ompD*, and *ompF*), leading to increased antibiotic susceptibility of *Salmonella typhimurium* LT2 [136].

The ferric catecholate receptor (FepA) on the surface of *Salmonella enterica* mediates iron transport and can adsorb phage H8. FepA mutant strains that are resistant to phage infection lose the ability to transport iron from the environment and die due to growth restriction.  The trade-off between phage resistance and growth restriction could be a useful phenomenon in phage therapy against MDR-*S.* *enterica* [137].
